# Risk of head and neck cancer in patients with peptic ulcers and the effect of *Helicobacter pylori* treatment

**DOI:** 10.1038/s41598-021-85598-4

**Published:** 2021-03-18

**Authors:** Yen-Ting Lu, Chung-Han Hsin, Ying-Chou Lu, Meng-Che Wu, Jing-Yang Huang, Cheng‐Chen Huang, Po-Hui Wang, Shun-Fa Yang

**Affiliations:** 1 Department of Otolaryngology, St. Martin De Porres Hospital, Chiayi, Taiwan; 2 Department of Otolaryngology, Chung Shan Medical University Hospital, Taichung, Taiwan; 3 Institute of Medicine, Chung Shan Medical University, No.110,Sec.1,Jianguo N.Rd., Taichung City, 40201 Taiwan; 4 Division of Gastroenterology, Children’s Medical Center, Taichung Veterans General Hospital, Taichung, Taiwan; 5 Center for Health Data Science, Chung Shan Medical University Hospital, Taichung, Taiwan; 6 Department of Obstetrics and Gynecology, Chung Shan Medical University Hospital, Taichung, Taiwan; 7 Department of Medical Research, Chung Shan Medical University Hospital, Taichung, Taiwan

**Keywords:** Cancer, Drug discovery, Diseases, Gastroenterology, Health care, Health occupations, Medical research, Risk factors

## Abstract

It remained inconclusive whether patients with peptic ulcer disease had a higher risk of head and neck cancer (HNC). Therefore, we enrolled 109,360 patients with peptic ulcer disease and matched for age and sex with 218,720 controls from the Taiwan National Health Insurance Research Database between January 1, 1997 and December 31, 2013.The HNC incidence rate was 1.33-fold higher in the peptic ulcer group than in the control group (7.52 vs. 5.68 per 100,00 person-years; crude relative risk: 1.33; 95% confidence interval [CI]: 1.08–1.63) after > 6 years of follow-up. However, in the peptic ulcer subgroup with *H. pylori* treatment, HNC risk was not significantly different from that of the control group (crude relative risk: 1.12; 95% CI: 0.86–1.46). Moreover, the population with peptic ulcers had the highest risk of laryngeal and hypopharyngeal cancer (adjusted HR: 2.27 [95% CI: 1.16–4.44] and 2.00 [95% CI, 1.13–3.55]), respectively. This observational study suggested that peptic ulcer disease is associated with an increased incidence of laryngeal and hypopharyngeal cancer and *H. pylori* treatment may have a role in preventing HNC in patients with peptic ulcer disease.

## Introduction

*Helicobacter pylori*, a spiral-shaped gram-negative bacterium in the aerodigestive tract, is a major cause of peptic and duodenal ulcers^1–8^. In addition to the lower aerodigestive tract, *H. pylori* can immigrate to the upper aerodigestive zone^9^. The human stomach is not the only reservoir of *H. pylori*, and the bacteria can be observed in the dental plaque, saliva, tonsils, and even adenoid tissue related to gastroesophageal reflux^10^.

The International Agency for Research on Cancer Working Group (IARC-1994) reported that *H. pylori* causes gastric cancer and lymphoma^3, 11^. Because *H. pylori* can immigrate to the upper aerodigestive zone, several studies have discussed the association between laryngeal or hypopharyngeal cancer and *H. pylori*^12–21^. However, results have been inconsistent, and most studies have been single-institute, cross-sectional, or case–control studies rather than long-term cohort studies^12–21^. Furthermore, *H. pylori* treatment was associated with a decreased incidence of gastric cancer in a systematic review and meta-analysis^22^. Currently, no study has discussed whether *H. pylori* treatment can reduce the incidence of head and neck cancer (HNC).

Taiwan is a newly industrialized country with a rapidly aging population. The prevalence of *H. pylori* in Taiwan is as high as 50–60%, providing a suitable population for evaluating the relationship between *H. pylori* and HNC^7, 8^. To address the aforementioned research gap, this nationwide population base cohort study investigated (1) whether peptic ulcer disease is a risk factor for HNC and (2) whether *H. pylori* treatment can reduce the risk of HNC in patients with peptic ulcer disease.

## Materials and methods

### Data source

The National Health Insurance (NHI) program is a single-payer, compulsory, universal health insurance plan established on March 1, 1995 that covers all forms of health care services for more than 99% of Taiwan’s 23.5 million residents^23, 24^. The National Health Insurance Research Database (NHIRD) included comprehensive medical data, including records of registration, ambulatory and inpatient care, catastrophic illnesses, and drug prescriptions^23–27^. Patient identification numbers and other sensitive personal data have been encrypted in NHIRD to protect individual privacy^27^. This study used data from the Longitudinal Health Insurance Database 2000 (LHID 2000), which is a subset of the NHIRD. The LHID 2000 contains detailed information of 1 million randomly selected patients from the 2000 Registry of Beneficiaries of the NHIRD by using a systematic sampling method; moreover, this subset contains all claims data recorded between 1997 and 2013^28^. Data in the LHID 2000 exhibit identical sex and age distribution to that in the NHIRD; therefore, the LHID 2000 is representative of the national patient population^29, 30^.

This study was approved by the Institutional Review Board of St. Martin De Porres Hospital and waived the requirement for patients’ informed consent as part of the study approval. All methods were carried out in accordance with relevant guidelines and regulations. Diagnostic codes in this study were defined using the *International Classification of Diseases, Ninth Revision, Clinical Modification* (ICD-9-CM).

### Study design and sample cohort generation

This research was designed to analyze the relationship between *H. pylori* treatment and subsequent HNC in a cohort of patients with peptic ulcer disease. Patients diagnosed as having various types of peptic ulcers (ICD-9 codes 531-533) and receiving treatment involving proton pump inhibitors (PPIs) between January 1, 1997, and December 31, 2013, were selected from the LHID 2000. PPI treatment was defined as the usage of omeprazole, pantoprazole, lansoprazole, rabeprazole, and esomeprazole. Furthermore, all included patients made at least 2 outpatient service claims or were hospitalized at least once and received a subsequent diagnosis of peptic ulcer disease during the designated period to increase the positive predictive value^31^. Patients were excluded if they had undergone *H. pylori* treatment before receiving a diagnosis of peptic ulcers or if they received a diagnosis of HNC or died before the index date. The index date was defined as 90 days after the first diagnosis of peptic ulcer disease. The comparison group (2 patients for every 1 patient with peptic ulcer) was selected using propensity score matching (PSM) for age and sex. All patients in this study were followed until the end of the study period (December 31, 2013) or their voluntary withdrawal from the NHI Program^23, 32, 33^.

Patients with peptic ulcer were categorized into 2 subgroups on the basis of *H. pylori* treatment, which was defined as (1) the addition of clarithromycin or metronidazole treatment for 7–14 days within 28 days of PPI treatment, (2) the addition of amoxicillin or tetracycline treatment for 7–14 days within 28 days of PPI treatment, or (3) the combination of the 2 aforementioned with the addition of bismuth for 7–14 days.

### Outcome and covariate measurement

The primary outcome in this study was a new diagnosis of HNC. End of the study period (December 31, 2013), voluntary withdrawal from the program, and death were defined as censoring events in this study. The HNC diagnosis was subgrouped on the basis of the following original recorded cancer sites: oral cancer (ICD-9-CM codes 140-145), laryngeal cancer (ICD-9-CM code 161), oropharyngeal cancer (ICD-9-CM code146), hypopharyngeal cancer (ICD-9-CM code148), nasopharyngeal cancer (NPC, ICD-9-CM code147) and nasal and sinus cancer (ICD-9-CM codes160.0-160.9 except 160.1).

Comorbidities were defined using ICD diagnostic codes as at least 1 hospital admission or 2 outpatient visits to treat a given disease within 2 years before the index date^34^. To increase the representative accuracy of our study, the following comorbid diseases were considered: hypertension (ICD-9 codes 401–405), diabetes mellitus (DM, ICD-9-CM code 250), asthma (ICD-9-CM code 493), chronic obstructive pulmonary disease (COPD, ICD-9-CM codes 490-492 and 493-496), chronic kidney disease (ICD-9-CM code 585), and chronic liver disease (ICD-9-CM codes 571 and 573). Most of these comorbidities were validated in a previous NHIRD study^33, 35^.

### Statistical analysis

PSM was performed for the peptic ulcer and control groups to ensure that baseline characteristics (sex, age, urbanization, length of hospital days, baseline co-morbidity) did not differ significantly between the groups with a standardized difference (SD) of < 0.1^36, 37^. The HNC incidence rate per 10,000 person-years was obtained by dividing the number of patients with HNC by the total person-months at risk. The log-rank test and Kaplan–Meier curves were used to evaluate the cumulative incidence of HNC between the groups. Adjusted hazard ratios (aHRs) for HNC occurrence in each group were estimated using a multiple Cox proportional hazards model. All statistical analyses were conducted using SAS 9.4 (SAS Inc., Cary, NC, USA), and two-sided *P* < 0.05 was considered statistically significant.

## Results

### Demographic characteristics

**Figure 1:**
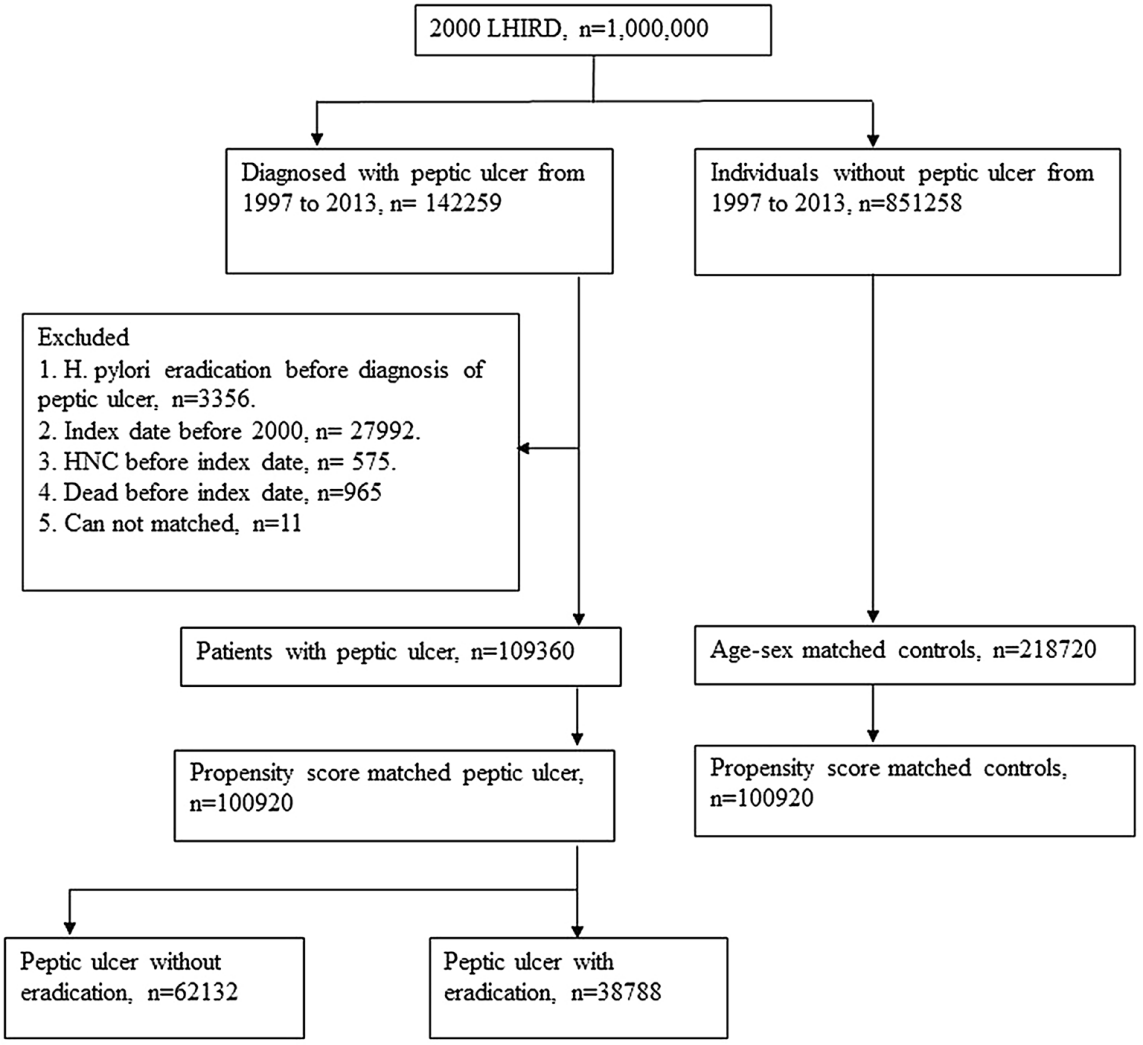
Flow diagram of identification and enrollment of the study patients. LHID = Longitudinal Health Insurance Database; *H. pylori*., *Helicobacter pylori*; HNC = Head and Neck Cancer.

This study included data of 109,360 patients with peptic ulcers and 218,720 controls matched at a 1:2 ratio for age and sex without peptic ulcers (Fig. 1). After PSM to eliminate different baseline characteristics in both the peptic ulcer and comparison groups, each group had 100,920 individuals (Supplementary Table 1). In the peptic ulcer group, 62,132 patients did not undergo *H. pylori* treatment, and 38,788 patients underwent *H. pylori* treatment.

The HNC incidence rate in the peptic ulcer and control groups is presented in Table 1. The overall (0–14 years) incidence density rate (per 10,000 person-years) of HNC in the total peptic ulcer and control groups were 7.31 and 6.59, respectively. Although the incidence rate was higher in the total peptic ulcer group, no significant difference was observed between the groups; the crude relative risk was 1.11 (95% confidence interval [CI]: 0.99–1.25). Then, the overall period was divided into 2 groups according to the follow-up periods of 0 to 6 years and > 6 years. In the > 6 years follow up, the incidence of HNC in the control, and total peptic ulcer were 5.68, 7.52, respectively. Compared with the control group, the crude relative risks (95% CIs) of HNC in the total peptic ulcer is significant higher 1.33 (1.08–1.63).

**Table 1:** Incidence per 10,000 person-years and crude relative risk for head and neck cancer during 14 years follow-up period.

	Control n = 100,920	Peptic ulcer		
		Total n = 100,920	Untreated n = 62,132	Treated n = 38,788
**Overall period (0–14 years)**				
Observed HNC case	512	584	377	207
Incidence density^a^ (95% C.I.)	6.59(6.04–7.18)	7.31(6.73–7.92)	8.04(7.27–8.90)	6.25(5.46–7.16)
Unadjusted hazard ratio (95% C.I.)	Reference	1.11(0.99–1.25)	1.22(1.07–1.40)	0.95(0.81–1.12)
Cumulative incidence^b^ at 3 years (95% C.I.)	2.01(1.74–2.32)	2.09(1.82–2.41)	2.36(1.99–2.80)	1.67(1.30–2.14)
Cumulative incidence^b^ at 6 years (95% C.I.)	4.29(3.86–4.77)	4.30(3.88–4.77)	4.66(4.10–5.30)	3.75(3.15–4.46)
Cumulative incidence^b^ at 9 years (95% C.I.)	5.72(5.19–6.29)	6.74(6.16–7.37)	7.54(6.75–8.43)	5.59(4.80–6.51)
Cumulative incidence^b^ at 12 years (95% C.I.)	7.69(6.99–8.45)	8.79(8.05–9.60)	9.75(8.73–10.88)	7.44(6.42–8.63)
**0–6 years**				
Observed HNC case	353	363	237	126
Incidence density^a^ (95% C.I.)	7.10(6.40–7.88)	7.18(6.47–7.96)	7.84(6.90–8.90)	6.19(5.20–7.37)
Unadjusted hazard ratio (95% C.I.)	Reference	1.03(0.89–1.19)	1.10(0.94–1.30)	0.87(0.71–1.07)
**> 6 years**				
Observed HNC case	159	221	140	81
Incidence density^a^ (95% C.I.)	5.68(4.86–6.62)	7.52(6.60–8.59)	8.42(7.14–9.94)	6.36(5.11–7.91)
Unadjusted hazard ratio (95% C.I.)	Reference	1.33(1.08–1.63)	1.49(1.18–1.86)	1.12(0.86–1.46)

PSM = propensity score matched; HNC = head and neck cancer.

^a^Incidence density rate, per 10,000 person-years.

^b^Cumulative incidence probability, per 1000 person.

**Figure 2:**
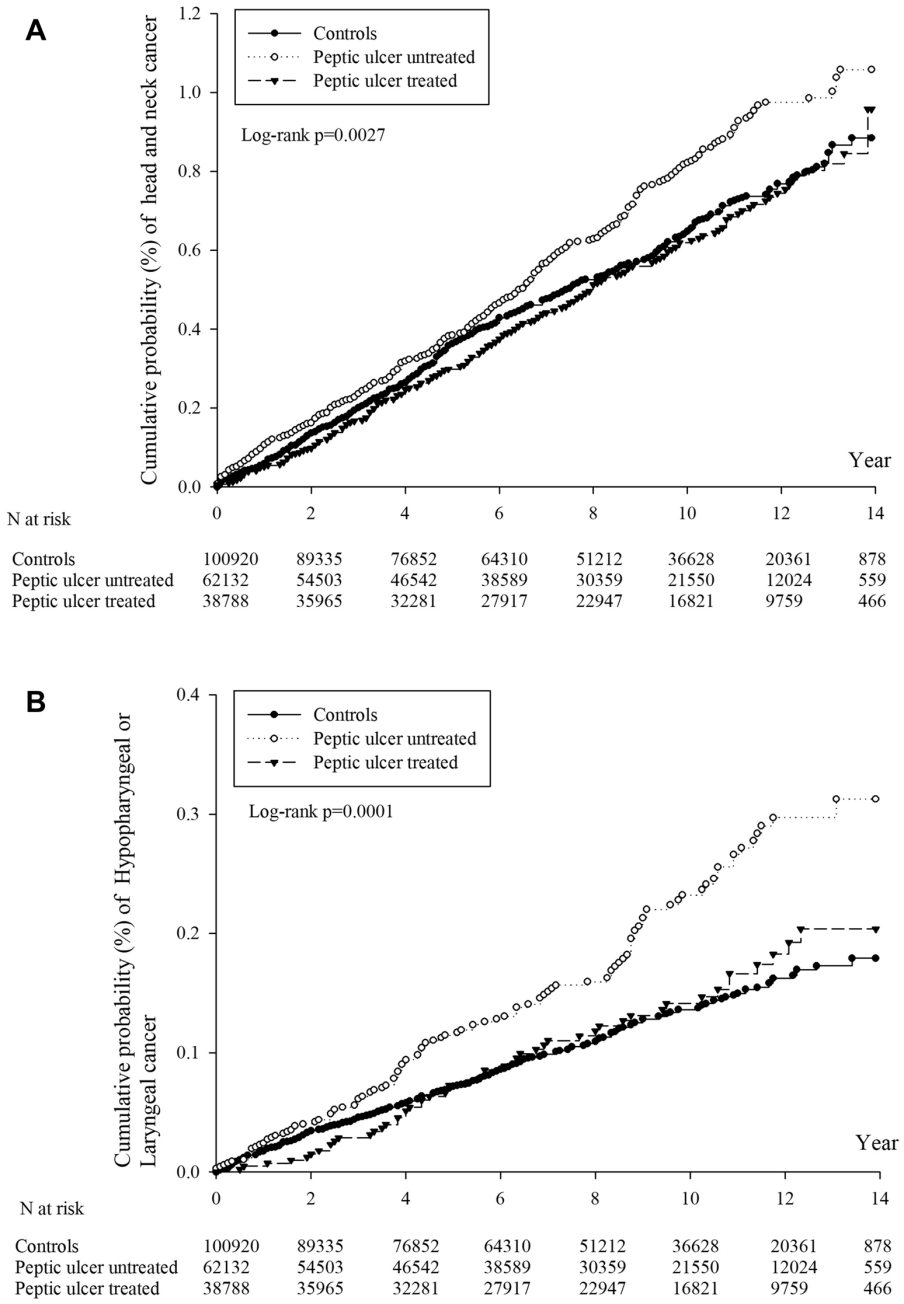
(**A**) The cumulative incidence functions of HNC for peptic ulcer without eradication, peptic ulcer with eradication and controls. (**B**) The cumulative incidence functions of hypopharyngeal/laryngeal cancer for peptic ulcer without eradication, peptic ulcer with eradication and controls.

Total peptic ulcer group was divided to 2 groups as “with *H. pylori* treatment” and “without *H. pylori* treatment” to eliminate its possible effect of HNC risk. The incidence density of HNC for the overall period in the groups with and without *H. pylori* treatment was 6.25 and 8.04, respectively. The crude relative incidence of HNC in the groups with and without H. pylori treatment was 0.95 (95% CI: 0.81–1.12) and 1.22 (95% CI: 1.07–1.40) compared with the control group. In > 6 years follow-up period, the incidence of HNC for the overall period in the groups with and without *H. pylori* treatment can be even upto and 8.42 and 6.36, respectively Then, the crude relative incidence of HNC in the groups with and without *H. pylori* treatment was 1.12 (95% CI: 0.86–1.46) and 1.49 (95% CI: 1.18–1.86) compared with the control group. The cumulative probabilities of HNC in the PSM population are represented as Kaplan–Meier curves in Fig. 2A (log-rank test, *P* = .0027).

The effects of peptic ulcer disease, *H. pylori* treatment, and covariates on HNC were assessed using the multiple Cox proportional hazards model. Results are presented in Table 2. A significantly different crude relationship emerged between peptic ulcer disease and HNC when follow-up time was > 6 years (aHR: 1.24; 95% CI: 1.01–1.51). Furthermore, when comparing *H. pylori* treatment policies with the control group, the group without treatment exhibited a significantly higher incidence of HNC than did the control group in the overall period (aHR: 1.23; 95% CI: 1.08–1.40) and after > 6 years of follow-up (aHR: 1.44 95%; CI: 1.16–1.80). However, the incidence of HNC in the group receiving *H. pylori* treatment was not significantly different from that of the control group in the overall period and the follow-up period of > 72 months. Additionally, sex, age, urbanization, length of hospital stay, and chronic liver disease were possible factors related to HNC. A sensitivity analysis was then performed to adjust possible effects influencing HNC incidence because of differences in follow-up time, sex, and age. The peptic ulcer group without treatment still exhibited a higher incidence of HNC compared with the control group with significant differences in different subgroups (sex and age). The peptic ulcer group with treatment exhibited the lowest incidence of HNC.

**Table 2:** Multiple Cox proportional hazard regression for estimation of adjusted hazard ratios on head and neck cancer.

Variable	Model for peptic ulcer		Model for eradication	
	Overall period	≥ 6 years	Overall period	≥ 6 years
**Study group (ref: control)**				
Peptic ulcer	1.09(0.96–1.22)	1.24(1.01–1.51)		
Peptic ulcer without eradication			1.23(1.08–1.40)	1.44(1.16–1.80)
Peptic ulcer with eradication			0.90(0.76–1.05)	1.01(0.78–1.31)
**Sex (ref: female)**				
Male	4.85(4.14–5.68)	5.08(3.9–6.61)	4.89(4.17–5.73)	5.06(3.90–6.57)
**Age (ref: 30–45)**				
< 30	0.22(0.15–0.33)	0.21(0.12–0.38)	0.22(0.14–0.32)	0.20(0.11–0.37)
45–65	1.58(1.35–1.84)	1.12(0.89–1.42)	1.58(1.36–1.84)	1.15(0.91–1.45)
≥ 65	1.30(1.07–1.58)	1.11(0.81–1.52)	1.28(1.06–1.56)	1.06(0.78–1.46)
**Urbanization (ref: Urban)**				
Sub-urban	1.17(1.02–1.33)	1.19(0.95–1.48)	1.16(1.02–1.33)	1.17(0.94–1.46)
Rural	1.39(1.17–1.66)	1.39(1.03–1.87)	1.38(1.15–1.65)	1.36(1.01–1.83)
**Length of hospital stays**^**a**^** (ref: 0 day)**				
1–6 days	1.24(1.05–1.47)	1.10(0.82–1.48)	1.24(1.04–1.46)	1.08(0.81–1.45)
≥ 7 days	1.57(1.32–1.86)	1.50(1.11–2.03)	1.53(1.29–1.81)	1.52(1.13–2.04)
**Baseline co-morbidity**				
Hypertension	1.06(0.92–1.22)	1.06(0.83–1.36)	1.05(0.91–1.21)	1.09(0.86–1.40)
Diabetes mellitus	1.08(0.91–1.28)	1.04(0.76–1.41)	1.08(0.91–1.27)	0.99(0.73–1.34)
Asthma	0.94(0.73–1.20)	1.00(0.65–1.52)	0.93(0.73–1.20)	1.04(0.69–1.56)
COPD	1.01(0.85–1.20)	0.90(0.67–1.21)	1.01(0.85–1.19)	0.92(0.69–1.23)
Chronic kidney disease	1.06(0.69–1.62)	0.38(0.09–1.52)	1.04(0.68–1.60)	0.36(0.09–1.44)
Chronic liver diseases	1.39(1.22–1.58)	1.27(1.01–1.59)	1.38(1.21–1.58)	1.29(1.04–1.62)

COPD, chronic obstructive pulmonary disease.

^a^Length of hospital stays, was the sum of the days in the hospitalization within 2 years before index date.

We subgrouped HNC on the basis of its origins as follows: oral cancer, oropharyngeal cancer, NPC, laryngeal cancer, and nasal/sinus cancer. We analyzed aHRs for HNC incidence by origin between the peptic ulcer and control groups by using a multiple Cox proportional hazards model. The peptic ulcer group had a significantly higher incidence of hypopharyngeal (aHR: 2.004; 95% CI: 1.130–3.551) and laryngeal cancer (aHR: 2.272; 95% CI: 1.163–4.440) compared with the control group after > 6 years of follow-up (Table 3). Furthermore, the peptic ulcer group without treatment had the highest incidence of hypopharyngeal and laryngeal cancer with aHR (95% CI) reaching 2.790 (1.525–5.104) and 2.858 (1.403–5.824), respectively. However, the HNC incidence in the group with treatment did not differ from that in the control group for all HNC origins. The cumulative probabilities of hypopharyngeal and laryngeal cancer were significantly different among the peptic ulcer with treatment, peptic ulcer without treatment, and control groups (log-rank test, *P* < .0001), as revealed by Kaplan–Meier curves (Fig. 2B). Otherwise, in order to eliminate the possible cofactors related to HNC in different groups, we arranged the stratified analyses by age, sex, urbanization, and hospitalization at baseline in Table 4. In the sensitivity analysis among all subgroups, the population with untreated peptic ulcer had a significant higher risk of HNC comparing with control group especially in the subgroups with characters as male, < 30 years old, urban and hospitalization at baseline with aHR (95% CI) reaching 1.23(1.07–1.43), 2.57(1.13–5.89), 1.44(1.20–1.72) and 1.60(1.27–2.01), separately.

**Table 3:** Adjusted hazard ratio of different origin of head and neck caner (Follow up > 6 years).

Sub-events	Control	Peptic ulcer		
		Total	Without eradication	With eradication
Laryngeal cancer	Reference	2.272(1.163–4.440)	2.858(1.403–5.824)	1.539(0.647–3.661)
Hypopharyngeal cancer	Reference	2.004(1.130–3.551)	2.790(1.525–5.104)	1.121(0.512–2.450)
Oral cancer	Reference	1.154(0.887–1.500)	1.352(1.006–1.816)	0.922(0.650–1.308)
Oropharyngeal cancer	Reference	1.383(0.794–2.407)	1.502(0.799–2.824)	1.246(0.623–2.492)
Nasopharyngeal cancer	Reference	0.995(0.641–1.543)	0.940(0.552–1.600)	1.060(0.617–1.820)
Nasal/sinus cancer	Reference	0.795(0.288–2.193)	0.787(0.236–2.620)	0.805(0.213–3.047)

**Table 4:** The sensitivity analysis for the adjusted hazard ratio (95% C.I.) stratified by follow up time, sex and age groups after PSM.

	aHR (95% C.I.) of HCN			
	Control	Peptic ulcer		
		Total	Untreated	Treated
**Overall follow up time**				
Sex subgroups				
Female	Reference	1.01(0.76–1.35)	1.20(0.87–1.64)	0.71(0.46–1.11)
Male	Reference	1.10(0.97–1.25)	1.23(1.07–1.43)	0.93(0.78–1.11)
Age at index date				
< 30 years old	Reference	1.94(0.86–4.35)	2.57(1.13–5.89)	0.68(0.15–3.13)
30–44 years old	Reference	1.16(0.91–1.49)	1.35(1.02–1.78)	0.95(0.68–1.32)
45–64 years old	Reference	1.13(0.96–1.33)	1.29(1.07–1.55)	0.95(0.76–1.18)
≥ 65 years old	Reference	0.89(0.69–1.13)	0.96(0.73–1.25)	0.74(0.51–1.08)
Urbanization				
Urban	Reference	1.21(1.03–1.43)	1.44(1.20–1.72)	0.94(0.76–1.17)
Sub-urban	Reference	0.96(0.78–1.19)	1.04(0.82–1.32)	0.86(0.65–1.14)
Rural	Reference	0.93(0.68–1.28)	1.00(0.70–1.42)	0.83(0.53–1.29)
Hospitalization at baseline				
No	Reference	0.97(0.84–1.12)	1.07(0.90–1.26)	0.85(0.71–1.03)
Yes	Reference	1.39(1.12–1.72)	1.60(1.27–2.01)	1.00(0.73–1.38)
**Follow up > 6 years**				
Sex subgroups				
Female	Reference	1.12(0.69–1.81)	1.31(0.77–2.22)	0.85(0.43–1.69)
Male	Reference	1.27(1.02–1.58)	1.48(1.15–1.89)	1.03(0.77–1.37)
Age at index date				
< 30 years old	Reference	1.39(0.44–4.39)	1.88(0.58–6.18)	0.54(0.06–4.63)
30–44 years old	Reference	1.54(1.07–2.23)	1.86(1.24–2.80)	1.21(0.76–1.93)
45–64 years old	Reference	1.14(0.85–1.53)	1.37(0.98–1.91)	0.91(0.62–1.34)
≥ 65 years old	Reference	1.07(0.69–1.68)	1.11(0.68–1.81)	1.02(0.55–1.86)
Urbanization				
Urban	Reference	1.29(0.98–1.69)	1.59(1.17–2.16)	0.95(0.66–1.37)
Sub-urban	Reference	1.19(0.84–1.69)	1.37(0.92–2.02)	0.97(0.61–1.55)
Rural	Reference	1.20(0.71–2.05)	1.13(0.61–2.10)	1.31(0.68–2.54)
Hospitalization at baseline				
No	Reference	1.19(0.94–1.50)	1.38(1.06–1.80)	0.98(0.73–1.33)
Yes	Reference	1.37(0.94–2.02)	1.60(1.05–2.44)	1.04(0.61–1.79)

PSM, propensity score matched; HNC, head and neck cancer.

## Discussion

According to our literature review, this is the first study to investigate the association between peptic ulcer disease and HNC risk as well as the possible effects of *H. pylori* treatment. We observed an association between peptic ulcer disease and an increased incidence of laryngeal and hypopharyngeal cancer after follow-up for more than 6 years. Specifically, among patients with peptic ulcer disease, *H. pylori* treatment significantly reduced the risk of HNC compared with those without *H. pylori* treatment. Furthermore, HNC incidence in the peptic ulcer group with *H. pylori* treatment could be as low as that in healthy patients without peptic ulcers.

The relationship between peptic ulcer disease and HNC is crucial because of their complications and substantial effects on quality of life. Kuipers et al. demonstrated that *H. pylori* infection was diagnosed in as many as 60–100% patients with peptic ulcer disease^38^. This relationship between peptic ulcer disease and HNC can be largely attributed to the relationship between *H. pylori* and HNC. However, few studies have addressed the potential role of *H. pylori* infection in laryngohypopharyngeal carcinoma and have had with conflicting results^13, 15, 16, 19–21, 39, 40^. Rezaii et al. evaluated *H. pylori* serology in 70 cases of laryngeal carcinoma, 28 cases of hypopharyngeal carcinoma, and 105 healthy controls^20^. After a multivariate regression to eliminate other confounding factors, the odds ratio of *H. pylori* seropositivity reached 11.49 in the laryngopharyngeal cancer group^20^. However, Morand et al. performed serological tests, rapid urease tests, and quantitative polymerase chain reactions (qPCRs) for 56 patients with HNC and 90 cancer-free controls. In a logistic regression analysis, the rates of neither positive serology nor rapid urease tests were significantly different between the 2 groups (*P* = .677 and *P* = .633, respectively)^19^. Both of these studies were case–control studies with limited case numbers in a single institute. Moreover, they focused on the difference in *H. pylori* prevalence between patients with HNC and those without cancer. Cohort studies with longitudinal follow-up and large number big data are lacking.

We enrolled 142,259 patients with peptic ulcer disease and 218,720 age- and sex-matched controls from the LHID 2000. After PSM to eliminate possible confounding factors, the peptic ulcer and control groups each included 100,920 cases (Table 1). Incidences of HNC (follow-up period > 6 years) were 5.68, 7.52, 8.42, and 6.36 per 10,000 person-years in the control, total peptic ulcer, peptic ulcer without treatment, and peptic ulcer with treatment groups, respectively after PSM. The crude relative risk (95% CI) of the peptic ulcer group reached 1.33 (1.08–1.63) and that of the peptic ulcer subgroup without *H. pylori* treatment reached an even higher 1.49 (1.18–1.86). However, in the group with *H. pylori* treatment, HNC incidence did not differ significantly from that of the control group (crude relative risk: 1.12; 95% CI: 0.86–1.46). The results of this study also revealed that laryngeal and hypopharyngeal cancer were the 2 HNCs most associated with peptic ulcer disease. The aHRs for hypopharyngeal and laryngeal cancer were 2.004 and 2.272, respectively, in the peptic ulcer group with a follow-up period of > 6 years (Table 3). Otherwise, to eliminate the possible cofactors related to HNC, we arranged sensitivity analysis (Table 4). The results revealed that the population with untreated peptic ulcer has a higher risk of HNC comparing with control group in different age, sex or hospitalization at baseline subgroups (Table 4). Especially in the population with factors as male, < 30 years old, urban and hospitalization at baseline, the aHR (95% CI) can reach 1.23(1.07–1.43), 2.57(1.13–5.89), 1.44(1.20–1.72) and 1.60(1.27–2.01), separately. Hospitalization at baseline revealed that these patients had admitted to hospital for further treatment due to severe peptic ulcer or associated complications such as massive bleeding. Therefore, population with hospitalization at base could be regarded as having more severe peptic ulcer or poor general condition comparing with those without hospitalization at baseline. Therefore, *H. pylori* treatment could play important role for prevention of HNC especially when patients with severe peptic ulcer or having factors as male, < 30 years old, or urban.

*Helicobacter pylori* is a microaerophilic gram-negative bacterium discovered by Marshall and Warren in 1984^1^ that affects the etiopathogenesis of chronic illnesses and causes cancer in digestive regions^41–43^. The mechanism by which *H. pylori* induces gastric cancer is related to its characteristics of causing persistent inflammation and the development of epithelial metaplasia and genetic instability in gastric regions^44^. Squamous cell carcinoma, which is a dominant cancer type in the head and neck region, is also related to abnormal epithelial proliferation and differentiation. In addition to the stomach, the oral cavity, tonsil tissue, and saliva are reservoirs of *H. pylori*, which can colonize in the head and neck region via oral and gastric oral routes (gastroesophageal reflux)^40^. Therefore, *H. pylori* induces epithelial cell proliferation in the laryngeal mucosa, as it does in the gastric mucosa, eventually causing laryngeal carcinoma^12, 40^.

*H. pylori* treatment can reduce progressive inflammation, histologic damage to the gastric mucosa, peptic ulcer occurrence and recurrence, and the risk of gastric cancer^44^. In a nationwide population-based cohort study of Taiwan, Wu et al. demonstrated that the incidences of gastric ulcer diseases decreased by 42–48% after *H. pylori* treatment and PPI use between 1997 and 2006^45^. In addition, a meta-analysis by Lee et al. enrolled 20,484 patients with *H. pylori* treatment and 27,580 patients without *H. pylori* treatment^46^. After a total follow-up of 340,255 person-years, the authors revealed a significant reduction in gastric cancer risk by approximately 50%^46^. Thus, we hypothesized that *H. pylori* treatment can also reduce progressive inflammation and damage to the laryngeal and pharyngeal mucosa caused by *H. pylori* that may be related to laryngeal and hypopharyngeal cancer formation. Our results supported this hypothesis that after patients with peptic ulcer disease undergo *H. pylori* treatment, the risk of HNC can be similar to that in the population without peptic ulcer (crude relative risk: 1.12; 95% CI: 0.86–1.46). However, patients who did not undergo *H. pylori* treatment had a 1.49-fold higher risk of HNC compared with the population without peptic ulcer (crude relative risk: 1.49; 95% CI: 1.18–1.86).

This study has several unique strengths. First, this study included large study and control groups (109,360 patients with peptic ulcer and 218,720 age- and sex-matched controls) with long-term follow-up (14 years) by using nationwide insurance data (NHIRD). Because the NHI program provides information on 99% of Taiwan’s 23.5 million residents^23, 24^, we could trace nearly every patient with HNC within the designated period. Second, this was the first cohort study to evaluate the association among peptic ulcer disease, *H. pylori*, and HNC. Most studies have been cross-sectional studies evaluating the prevalence of *H. pylori* infection in HNC by using PCRs or other tests. Third, this is the first study to discuss the possible effect of *H. pylori* treatment in preventing HNC. Our results revealed that *H. pylori* treatment may have a role in HNC prevention.

This study has several limitations. First, diagnoses of peptic ulcer disease depended on ICD-9 codes recorded in the NHIRD and might have been inaccurate^33, 47^. Although we added inclusion criteria of (1) anti-acid medication use and (2) at least 2 outpatient department visits or 1 hospitalization to increase diagnostic precision, we lacked physical examination results and endoscopy findings^33, 47^. Second, information regarding smoking, alcohol intake, and betel nut use, which are crucial confounding factors of HNC, was unavailable in the NHIRD. Third, the proportion of *H. pylori* infections in the peptic ulcer group was unavailable.

## Conclusions

This is the first nationwide population-based cohort study to investigate the association between peptic ulcer disease and HNC as well as the possible role of *H. pylori* treatment. The risk of laryngeal and hypopharyngeal cancer was significantly higher in patients with peptic ulcer disease. However, *H. pylori* treatment successfully reduced the risk of HNC. On the basis of these results, physicians should consider *H. pylori* treatment when prescribing treatments for patients with peptic ulcers.

## Supplementary Information

Supplementary Table.
