# Supplementary material for: Risk of head and neck cancer in patients with peptic ulcers and the effect of Helicobacter pylori treatment
Source: Sci Rep. 2021 Mar 18;11:6229. doi: 10.1038/s41598-021-85598-4 (PMC7973765; doi:10.1038/s41598-021-85598-4)
Supplement: Supplementary file 1 — Supplementary Table. [file 41598_2021_85598_MOESM1_ESM.docx]

Supplementary Table 1. Baseline characteristics of study participants. PSM = propensity score matched; SD = standardized difference*Within 2 years before index date

|  | Before PSM | | | After PSM | | |
| --- | --- | --- | --- | --- | --- | --- |
|  | Control  n=218720 | Peptic ulcer  n=109360 | SD | Control  n=100920 | Peptic ulcer  n=100920 | SD |
| Sex |  |  | 0.00000 |  |  | 0.03212 |
| Female | 102916(47.05%) | 51458(47.05%) |  | 49386(48.94%) | 48307(47.87%) |  |
| Male | 115804(52.95%) | 57902(52.95%) |  | 51534(51.06%) | 52613(52.13%) |  |
| Age |  |  | 0.00000 |  |  | 0.00128 |
| <30 | 28874(13.2%) | 14437(13.2%) |  | 13559(13.44%) | 13603(13.48%) |  |
| 30-45 | 57496(26.29%) | 28748(26.29%) |  | 25562(25.33%) | 26629(26.39%) |  |
| 45-65 | 85348(39.02%) | 42674(39.02%) |  | 39127(38.77%) | 39170(38.81%) |  |
| >=65 | 47002(21.49%) | 23501(21.49%) |  | 22672(22.47%) | 21518(21.32%) |  |
| Urbanization |  |  | 0.02992 |  |  | 0.00934 |
| Urban | 131482(60.11%) | 64590(59.06%) |  | 59746(59.2%) | 60112(59.56%) |  |
| Sub-urban | 64723(29.59%) | 32567(29.78%) |  | 29971(29.7%) | 29867(29.59%) |  |
| Rural | 22515(10.29%) | 12203(11.16%) |  | 11203(11.1%) | 10941(10.84%) |  |
| Low income | 1234(0.56%) | 642(0.59%) | 0.00302 | 533(0.53%) | 571(0.57%) | 0.00511 |
| Length of hospital stays* |  |  | 0.43072 |  |  | 0.02422 |
| 0 day | 189214(86.51%) | 75718(69.24%) |  | 73801(73.13%) | 74232(73.56%) |  |
| 1-6 days | 16343(7.47%) | 15914(14.55%) |  | 14309(14.18%) | 13508(13.38%) |  |
| >=7 days | 13163(6.02%) | 17728(16.21%) |  | 12810(12.69%) | 13180(13.06%) |  |
| Baseline co-morbidity |  |  |  |  |  |  |
| Hypertension | 44670(20.42%) | 31007(28.35%) | 0.18545 | 28401(28.14%) | 27236(26.99%) | -0.02584 |
| Diabetes mellitus | 20589(9.41%) | 16345(14.95%) | 0.16978 | 14389(14.26%) | 13883(13.76%) | -0.01445 |
| Asthma | 9584(4.38%) | 7838(7.17%) | 0.11962 | 7102(7.04%) | 6629(6.57%) | -0.01861 |
| COPD | 19945(9.12%) | 16539(15.12%) | 0.18476 | 14725(14.59%) | 13979(13.85%) | -0.02117 |
| Chronic kidney disease | 2163(0.99%) | 2954(2.70%) | 0.12749 | 1860(1.84%) | 2039(2.02%) | 0.01289 |
| Chronic liver diseases | 19424(8.88%) | 26259(24.01%) | 0.41695 | 19048(18.87%) | 19674(19.49%) | 0.01575 |
